# Apolipoprotein C-III: Risk-factor, Regulator of Triglyceride-rich Lipoprotein Metabolism and Therapeutic Target

**DOI:** 10.1007/s11883-026-01399-y

**Published:** 2026-02-16

**Authors:** Elias Björnson, Martin Adiels, Marja-Riitta Taskinen, Chris J Packard, Jan Borén

**Affiliations:** 1 Department of Molecular and Clinical Medicine, Institute of Medicine, University of Gothenburg, Gothenburg, Sweden; 2 Research Program for Clinical and Molecular Metabolism, University of Helsinki, Helsinki, Finland; 3 Institute of Cardiovascular and Medical Sciences, University of Glasgow, Glasgow, UK; 4 Wallenberg Laboratory, Sahlgrenska University Hospital, Gothenburg, Sweden

**Keywords:** Apolipoprotein C-III, Triglyceride-rich lipoproteins, Hypertriglyceridemia, Lipoprotein metabolism, Lipoprotein lipase, Atherosclerosis

## Abstract

**Purpose of Review:**

Apolipoprotein C-III (apoC-III) has emerged as a pivotal regulator of triglyceride metabolism and a key factor in cardiovascular risk. This review explores the physiological and pathological roles of apoC-III, focusing on kinetic mechanisms, genetic data, and the therapeutic potential of targeting apoC-III.

**Recent Findings:**

Loss-of-function mutations in APOC3 significantly lower plasma triglyceride levels and coronary heart disease risk, validating apoC-III as a therapeutic target. Kinetic studies indicate that increased hepatic secretion of apoC-III raises triglyceride levels, particularly in individuals with type 2 diabetes. Beyond lipid metabolism, apoC-III promotes lipoprotein retention and amplifies arterial inflammation. Novel inhibitors, such as antisense oligonucleotides targeting APOC3, have been shown to markedly reduce plasma apoC-III and triglyceride concentrations in both preclinical and clinical studies.

**Summary:**

Genetic and mechanistic evidence together establish the inhibition of apoC-III as a promising strategy for patients at high risk of persistent hypertriglyceridemia and cardiovascular disease. ApoC-III not only controls lipid metabolism but also exerts direct pro-atherogenic and pro-inflammatory effects, supporting its role as a multifaceted therapeutic target in cardiometabolic medicine.

## Introduction

 Apolipoprotein C-III (apoC-III) has emerged as a key regulator of triglyceride metabolism and an important cardiovascular risk factor [1–3]. It is synthesised primarily in the liver and, to a lesser extent, in the intestine, where it is secreted on triglyceride-rich lipoproteins (TRL). Once in the bloodstream, apoC-III continuously moves between lipoprotein particles, including very low-density lipoproteins (VLDL), chylomicrons, low-density lipoproteins (LDL) and high-density lipoproteins (HDL) [3]. Although it was discovered over 50 years ago, apoC-III has only recently become recognised as a cardiovascular risk factor and potential therapeutic target following landmark genetic studies demonstrating that loss-of-function (LOF) mutations in the *APOC3* gene provide protection against cardiovascular disease [4, 5].

These studies demonstrated that individuals carrying rare *APOC3* LOF variants have substantially lower levels of plasma apoC-III and triglycerides, as well as improved HDL levels. They are also found to have a striking 40% reduction in coronary heart disease risk compared to non-carriers [5]. These findings established that lifelong deficiency of apoC-III leads to enhanced clearance of TRLs, greater lipolytic activity, and lower atherogenic burden, independent of traditional risk factors [6]. Importantly, the demonstration that *APOC3* LOF is associated with significant cardiovascular benefit has underpinned the development of apoCIII-inhibitory therapies, providing a strong genetic rationale for targeting *APOC3* to reduce residual cardiovascular risk [4, 7]. Consequently, the clinical development of antisense oligonucleotides and siRNA agents targeting apoC-III, such as volanesorsen, olezarsen and plozasiran [8–12], has the potential to transform the therapeutic landscape for patients with moderate and severe hypertriglyceridemia at risk of CHD [9, 10, 13], and has already proven effective in patients with extremely high triglyceride levels at risk of recurrent acute pancreatitis [8].

## Structure and Regulation of *APOC3* Expression

### Molecular Structure and Glycosylation

ApoC-III consists of 79 amino acid residues arranged into two amphipathic helices that mediate binding to lipoproteins [1, 5]. A unique feature of apoC-III is its post-translational modification through O-linked glycosylation at Thr-74, which results in three distinct glycoforms: apoC-III₀ (unsialylated), apoC-III₁ (monosialylated) and apoC-III₂ (disialylated) [14]. In healthy individuals, these glycoforms comprise 22%, 45% and 33% of total apoC-III, respectively [15].

The sialylation state of apoC-III appears to be functionally significant. Recent studies demonstrate that apoC-III₁ inhibits lipoprotein lipase (LPL) more effectively than apoC-III₂, despite the latter having a higher affinity for VLDL [16, 17]. Furthermore, the glycoforms exhibit differential clearance kinetics: apoC-III₂ is cleared preferentially by heparan sulfate proteoglycans (HSPGs), particularly syndecan-1; meanwhile, apoC-III₁ is cleared preferentially by LDL receptors (LDLRs) and LDL receptor-related protein 1 (LRP1) [18]. This differential clearance has therapeutic implications: treatment with volanesorsen (an apoC-III antisense oligonucleotide) increased the apoC-III₂/apoC-III₁ ratio by 40% by accelerating the clearance of apoC-III₁ [18].

Recent large-scale epidemiological research has revealed that the composition of circulating apoC-III glycoforms is related to vascular health, regardless of plasma triglyceride levels and conventional cardiometabolic risk factors. A prospective analysis of the Multi-Ethnic Study of Atherosclerosis (MESA) cohort revealed that higher baseline levels of glycosylated, non-sialylated apoC-III and lower levels of disialylated apoC-III, relative to monosialylated apoC-III, were associated with a slower decline in the ankle-brachial index, a surrogate for peripheral atherosclerosis, as well as a reduced risk of peripheral artery disease [19]. These relationships were observed across diverse ethnic groups and remained robust after adjusting for lipid concentrations and other confounders. Interestingly, total plasma apoC-III levels were not predictive of peripheral vascular outcomes, highlighting the importance of considering glycoform-specific effects [19]. These findings imply that the glycosylation status of apoC-III modulates vascular risk through mechanisms that extend beyond its recognised function in lipoprotein metabolism. Further investigation of these post-translational modifications could provide new insights into the prevention and treatment of atherosclerotic disease.

### Transcriptional Regulation

The expression of *APOC3* is regulated by nutritional and metabolic factors. Glucose stimulates *APOC3* expression via the carbohydrate-responsive element-binding protein (ChREBP) [20, 21] and the hepatic nuclear factor 4 alpha (HNF4alpha) [22], while insulin suppresses expression by phosphorylating the forkhead box protein O1 (FOXO1) [23]. This dual regulation creates a metabolic switch: in insulin-resistant states characterised by hyperglycaemia and impaired insulin signalling, *APOC3* expression increases, leading to elevated plasma apoC-III levels and hypertriglyceridaemia. Dietary factors also modulate apoC-III levels. Saturated fatty acids and fructose increase *APOC3* expression and plasma apoC-III concentrations [24], whereas polyunsaturated fatty acids, particularly omega-3 fatty acids, reduce apoC-III levels [25]. One notable example is a two-week intervention involving a low-carbohydrate, isocaloric diet (30 g of carbohydrates per day) for obese individuals with non-alcoholic fatty liver disease. This intervention resulted in a reduction of almost 50% in plasma apoC-III levels [26].

PPARα and PPARγ are key regulators of lipid metabolism that significantly influence *APOC3* expression [27]. This has a clinically relevant impact on the metabolism of TRLs and cardiovascular risk. Activation of PPARα, primarily by fibrates [28], has been shown to suppress *APOC3* transcription in hepatocytes by displacing HNF4α from its binding site in the *APOC3* promoter [29]. This leads to decreased plasma apoC-III levels and improved triglyceride clearance. PPARα binds directly to a specific site in the *APOC3* promoter (− 87/−66), thereby modulating gene transcription [30]. It may also act through the upregulation of Rev-erbα, which further represses *APOC3* expression [31]. However, the efficacy of PPARα agonists in lowering *APOC3* can be variable between individuals. In contrast, PPARγ primarily orchestrates adipocyte differentiation and the suppression of inflammatory genes, but also exerts secondary effects on hepatic lipid genes, including *APOC3*, via transcriptional co-regulators such as PGC-1β [27]. Elevated *APOC3* expression correlates with insulin resistance [32], and PPARγ agonists, such as thiazolidinediones, may indirectly influence hepatic *APOC3* regulation by improving systemic insulin sensitivity. Overall, PPARα and PPARγ are important but distinct pharmacological and physiological regulators of *APOC3* levels, with ongoing implications for novel therapies targeting hypertriglyceridaemia and cardiovascular risk. Clinical trials of PPARα and PPARγ agonists have yielded variable outcomes in terms of ASCVD prevention. The reason for the mixed results for fibrate therapy in particular is not entirely clear. Early studies were positive but the most recent trial [33] – PROMINENT – reported a null effect of pemafibrate on cardiovascular disease despite TRL lowering, presumably due in part to apoCIII reduction. Accordingly, the focus for therapeutic agents in development has shifted to drugs based on gene-silencing technologies with a well-defined mechanism of action and a direct effect on *APOC3* expression.

## ApoC-III Kinetic Studies

Stable isotope tracer kinetics have become the gold standard for investigating lipoprotein metabolism in humans. Studies typically involve the intravascular administration of deuterated leucine and glycerol as the tracers, and the collection of serial blood samples over periods ranging from 24 h to several days [34–36]. Multicompartmental modelling approaches are employed to derive kinetic parameters such as secretion rate (SR) and fractional catabolic rate (FCR) from the pool sizes and the tracer-tracer enrichment curves [34–36].

## Kinetic Findings in Type 2 Diabetes

Kinetic studies in subjects with type 2 diabetes have revealed that the SR of apoC-III is the primary determinant of plasma triglyceride levels [37]. One study, comparing subjects with type 2 diabetes to controls with a similar BMI, found that the diabetic group had a 39% higher apoC-III SR (676 ± 208 vs. 505 ± 174 mg/d, *P* = 0.042) and a 17% higher FCR [37].z Notably, the apoC-III SR was strongly correlated with plasma triglyceride levels (*r* = 0.9, *p* < 0.001), whereas the FCR showed no significant correlation [37].

Treatment with liraglutide (a glucagon-like peptide-1 analogue) for 16 weeks significantly reduced the apoC-III secretion rate by 14% (from 652 ± 196 to 561 ± 198 mg/day, *P* = 0.022) and plasma apoC-III levels by 14% in subjects with type 2 diabetes [37]. Significantly, reduction in the apoC-III SR correlated with improvement in glycemic control (*r* = 0.67, *P* = 0.009) and decrease in the post-prandial response to a test meal (plasma triglyceride area under the curve, *r* = 0.59, *P* = 0.025) [37]. These findings establish glucose homeostasis as a major regulator of apoC-III metabolism and identify apoC-III secretion rate as an important driver of TRL elevation in type 2 diabetes.

### Kinetic Studies with rare *APOC3* LOF Carriers

**Fig. 1:**
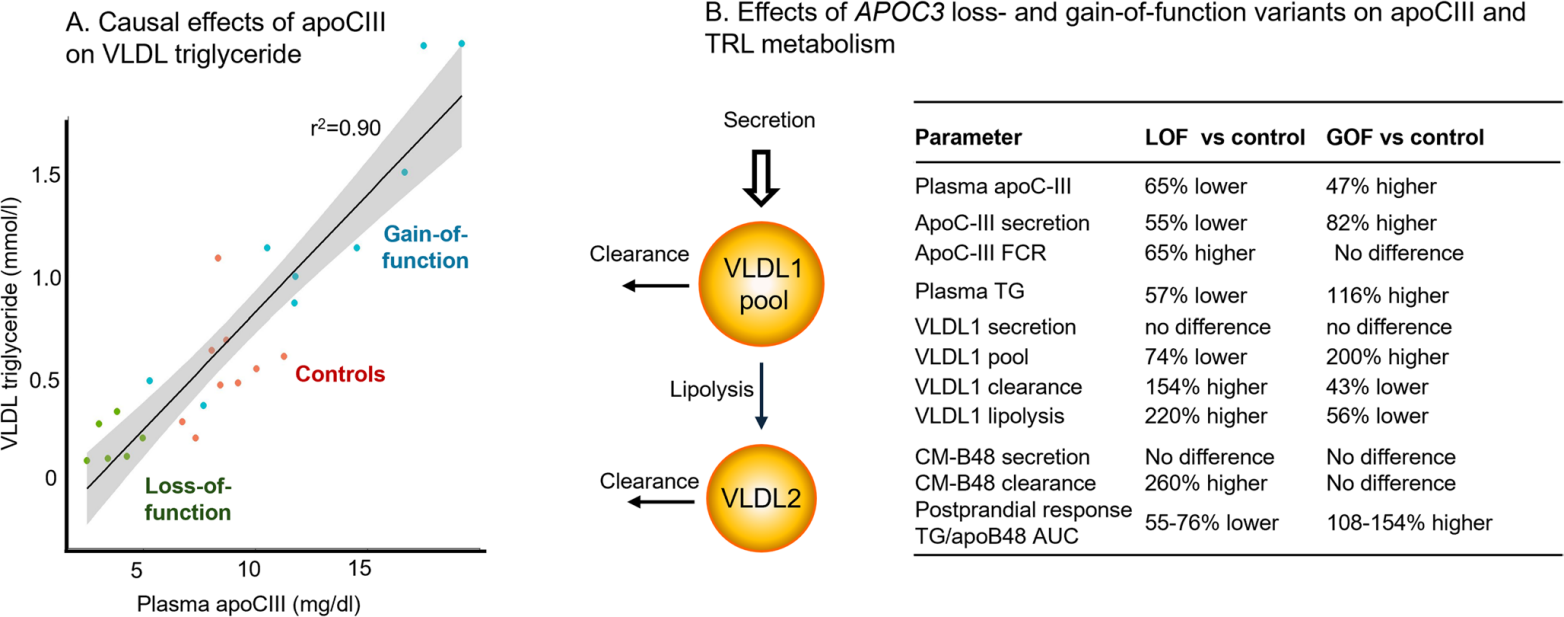
Metabolism of triglyceride-rich lipoproteins in subjects with loss-of-function and gain-of-function variants in apoC-III. By examining the metabolic consequences of genetic variation in *APOC3*, it is possible to establish the causal effects of the apoprotein on triglyceride transport and lipoprotein kinetics in general. (**A**) Association of plasma apoC-III concentration and VLDL triglyceride level across the 6 carriers of a *APOC3* LOF variant, 9 carriers of *APOC3* GOF variants, and 9 control subjects (carrying neither variant) (adapted from reference [41] with permission) [38, 41]. (**B**) Main differences in TRL apolipoprotein B metabolism between LOF carriers and controls and between GOF carriers and controls as reported in references [38, 41]. FCR=fractional catabolic rate; CM=chylomicrons; VLDL1, VLDL2 = very low density lipoprotein subfraction 1 and 2; AUC = area-under-curve; clearance = fractional clearance rate; lipolysis=fractional transfer rate.

Since plasma apoC-III levels are so strongly correlated with plasma TRL concentration and apoC-III circulates on TRL particles, it has been difficult until recently to establish categorically whether elevated apoC-III causes increased TRL, or vice versa. While drug studies such as those described above are highly supportive of the concept that apoC-III is a major regulator of TRL levels, the key information that apoC-III is a causal determinant of TRL metabolism has come from investigations of individuals with function-altering variants in the *APOC3* gene (Fig. 1). Studies of heterozygous carriers of *APOC3* loss-of-function (LOF) mutations have provided important insights into the physiological role of apoC-III [38, 39], and its relationship to atherosclerosis. LOF carriers were found to have markedly reduced coronary artery calcification scores [40]. Heterozygous carriers of the R19X null mutation have approximately 50% lower plasma apoC-III levels and 35% lower plasma triglyceride levels compared to non-carriers. Postprandial studies demonstrated dramatic effects on TRL metabolism: compared to controls (non-variant carriers). *APOC3* LOF carriers exhibited approximately threefold higher overall clearance rates for VLDL1, VLDL2, intermediate-density lipoprotein (IDL), and chylomicrons (Fig. 1) [38]. This resulted in substantially lower circulating levels of these lipoprotein classes, with the mean transit time for VLDL1 particles to become LDL falling from 17.7 h in non-carriers to 4.3 h in LOF carriers [38]. Importantly, however, chylomicron and VLDL production rates did not differ between LOF carriers and non-carriers, nor were LDL production or clearance rates affected [38].

### Kinetic Studies with *APOC3* Gain-of-function (GOF) Carriers

Recent studies of *APOC3* GOF variants (rs5128 and rs2854117) provide a mirror image of LOF findings (Fig. 1) [41]. GOF carriers had plasma apoC-III levels that were 47% higher as a result of increased apoC-III production rates compared to non-carriers [41]. The postprandial triglyceride response was 108% higher in GOF carriers due to higher VLDL1 levels. Kinetic analysis revealed lower FCR of 51% for VLDL1-apoB100 and 65% for intestinal-derived apoB48-containing VLDL1 in GOF carriers [41]. Interestingly, chylomicron metabolism appeared to be less affected, with no difference observed in the response of chylomicron apoB48 to a fat meal between GOF carriers and non-carriers. These findings suggest that genetically determined increases in apoC-III mainly affect TG transport in VLDL rather than in chylomicrons.

## Mechanisms of Action: How ApoC-III Regulates TRL Metabolism

### Inhibition of Lipoprotein Lipase

ApoC-III is a well-known inhibitor of lipoprotein lipase (LPL), the enzyme responsible for hydrolysing triglycerides in chylomicrons and VLDL [42]. This inhibition is achieved through multiple mechanisms. ApoC-III displaces apoC-II (an LPL activator) from lipoprotein surfaces and inhibits TRL binding to negatively charged cell surfaces where LPL resides. ApoC-III may also directly inhibit enzyme activity [42]. The aromatic tryptophan residues in the C-terminal half of apoC-III are particularly important for lipid binding and LPL inhibition [43]. The ratio of apoC-II to apoC-III on TRL surfaces determines the rate of lipolysis, with higher apoC-III/apoC-II ratios being associated with slower triglyceride hydrolysis [1, 42].

### Impaired Hepatic Clearance of Remnants

ApoC-III impairs the hepatic clearance of TRLs through pathways that are independent of LPL. This effect was strikingly demonstrated in patients with familial chylomicronaemia syndrome (FCS), which is caused by a genetic deficiency of LPL. Treatment with an antisense oligonucleotide that targets apoC-III, led to a significant reduction in serum triglyceride levels, confirming that apoC-III hinders remnant clearance beyond its role in lipolysis [44, 45]. The mechanisms involve interference with receptor-mediated uptake. ApoC-III displaces apoE from the surfaces of remnant particles, thereby preventing interaction with hepatic receptors, including LDLR, LRP1 and HSPGs. Studies in mice lacking both LDLR and LRP1 showed that treatment with apoC-III antisense oligonucleotides did not lower plasma triglycerides, confirming that these receptors are critical for the triglyceride-lowering effects of apoC-III inhibition. One model suggests that the metabolic impact of apoC-III depends on the presence of apoE. When apoE is present, apoC-III primarily suppresses hepatic remnant clearance. However, when apoE is limited or dysfunctional, apoC-III’s main effect shifts to inhibiting LPL. This property of apoC-III underpins the use of apoC-III inhibition as an effective preventive therapy for acute pancreatitis in individuals with complete or partial LPL deficiency (FCS and Multifactorial Chylomicronemis Syndrome (MCS)) [8].

### Effects on VLDL Assembly and Secretion

The role of apoC-III in VLDL assembly and secretion has been a matter of debate [46]. In vitro studies suggest that apoC-III enhances VLDL assembly and secretion under lipid-rich conditions by recruiting additional triglycerides to pre-VLDL particles [47]. However, human kinetic studies of both *APOC3* LOF and GOF carriers found no difference in VLDL production rates compared to non-carriers [38, 41]. This discrepancy suggests that the in vitro findings may not directly translate to the human in vivo situation. This is consistent with the notion that the primary effects of apoC-III in humans are on TRL clearance rather than production [46].

### Intestinal Metabolism

The impact of apoC-III on intestinal lipid metabolism varies significantly between experimental systems. In vitro studies using murine enteroids and Caco-2 cells demonstrate that intestinal *APOC3* expression is largely unresponsive to nutritional cues such as glucose, fatty acids, or insulin, reflecting an organ-specific regulatory profile distinct from the liver [48]. In mice, intestinal overexpression of apoC-III impairs chylomicron secretion and reduces lymphatic triglyceride output by inhibiting enterocyte lipid uptake and esterification, leading to lipid accumulation within the mucosa [49, 50]. Conversely, loss of intestinal apoC-III function or antisense oligonucleotide treatment paradoxically increases triglyceride secretion, indicative of enhanced lipid mobilization and export [51]. In humans, kinetic studies of *APOC3* LOF reveal that reduced apoC-III primarily accelerates postprandial clearance of TRLs without significantly altering their intestinal production, supporting a model where apoC-III modulates TRL processing rather than enterocyte lipid handling [38]. These findings underscore the limited translational relevance of rodent data and highlight complex, species-specific mechanisms governing intestinal lipid metabolism.

### ApoC-III Metabolism in Type 2 Diabetes, Chronic Kidney Disease, and Obesity

#### Type 2 Diabetes

Subjects with type 2 diabetes consistently have higher apoC-III SR and plasma levels than BMI-matched controls [37]. The main consequence of this apoCIII overproduction is, by retarding chylomicron and VLDL lipolysis and clearance, to drive up the number of TRL particles in the circulation. The particles, themselves, do not always show enrichment of apoC-III [52, 53].

Since apoC-III is regulated by both glucose via ChREBP and insulin via SREBP1, it’s overproduction may be regarded as a key component of diabetic dyslipidemia [54]. As such, apoC-III lowering is an important target to minimise ASCVD risk in this condition. Plasma levels of apoC-III in Type 2 diabetics may be reduced through improved glycaemic control, but there is an argument to go further and include apoCIII as part of the assessment of the plasma lipoprotein profile in subjects with Type 2 diabetes with a view to using *APOC3*-silencing agents when they become available.

#### Chronic Kidney Disease

Moderate chronic kidney disease (CKD) is associated with elevated plasma apoC-III due to impaired apoC-III catabolism (with FCR reduced by ~ 40%), rather than increased production [55]. The kidney plays a role in clearing free apoC-III from the blood, and impaired kidney function can lead to excess sialylation of apoC-III. This may make apoC-III-containing VLDL particles less susceptible for lipolytic degradation. ApoC-III FCR was strongly positively correlated with estimated glomerular filtration rate (*r* = 0.569, *P* < 0.01) [55].

#### Obesity and Metabolic Syndrome

These conditions are characterised by elevated apoC-III production rates and plasma levels [56, 57], with altered distributions of apoC-III glycoforms [58]. Weight loss through caloric restriction or bariatric surgery reduces the apoC-III₁/apoC-III₂ ratio, whereas feeding carbohydrates increases apoC-III₀ [59–61].

## Direct Atherogenic Effects Beyond Lipid Metabolism

### Lipoprotein Retention in the Artery Wall

ApoC-III increases the affinity of atherogenic lipoproteins for proteoglycans, facilitating their retention in the subendothelial space [62, 63]. Studies in people with type 2 diabetes show that LDL with high levels of endogenous apoC-III binds much more strongly to arterial wall proteoglycans than LDL with low levels of apoC-III [64]. However, direct enrichment with apoC-III in vitro only modestly increases binding, suggesting that other intrinsic features of diabetic LDL are critical for further enhancing proteoglycan affinity [64]. These changes are mechanistically closely linked to altered lipid composition: diabetic, apoCIII-rich LDL displays reduced levels of sphingomyelin, unesterified cholesterol, ceramide and GM1. This produces a more fluid lipid surface and exposes apoB100 binding domains that interact more avidly with proteoglycans [64]. ApoC-III also increases the susceptibility of LDL to hydrolysis and aggregation by arterial sphingomyelinases, which likely amplifies local inflammatory responses and atherogenesis [64]. Furthermore, a high level of apoC-III is associated with increased sialylation, and the disialylated apoC-III isoform (apoC-III_2_) on LDL is crucial for activating cellular inflammation, as demonstrated in vascular endothelial cell models [64]. Taken together, these findings suggest that apoCIII-enriched LDL is particularly susceptible to atherogenic modifications within the arterial intima, both through physical retention and enhanced susceptibility to enzymatic and inflammatory processes, which helps to explain the link between apoC-III and cardiovascular risk in diabetes [62].

### Proinflammatory and Prothrombotic Effects

ApoC-III activates adhesion molecules and proinflammatory responses directly in monocytes and endothelial cells. Studies have shown that apoC-III induces the expression of Vascular Cell Adhesion Molecule-1 (VCAM-1) in endothelial cells, thereby increasing monocyte adhesion via pertussis toxin-sensitive G protein and protein kinase C-α-mediated nuclear factor-κB activation [65, 66]. There is a strong correlation between plasma apoC-III levels and the activated factor VII-antithrombin (FVIIa-AT) complex, which is a biomarker for an increased predisposition to thrombotic events [67]. This association was observed in both sexes, regardless of prior history of coronary artery disease, suggesting that apoC-III links lipid metabolism to coagulation pathways.

### Pancreatic β-cell Dysfunction

Emerging research demonstrates that under conditions of islet insulin resistance, local pancreatic production of apoC-III plays a critical pathophysiological role by impairing β-cell function and contributing directly to β-cell failure in type 2 diabetes [68]. Elevated apoC-III expression in pancreatic islets mechanistically promotes a local inflammatory environment, increases mitochondrial metabolic activity, disrupts β-cell cytoplasmic calcium homeostasis and accelerates β-cell apoptosis [68]. These effects together lead to a progressive decline in insulin secretion capacity. This autocrine/paracrine loop is distinct from the hepatic and systemic roles of apoC-III, establishing the protein as a molecular link between insulin resistance and β-cell dysfunction. Notably, reducing islet apoC-III expression in experiments improves glucose tolerance and restores normal β-cell calcium handling without signs of local inflammation, highlighting the potential of targeting islet apoC-III therapeutically in diabetes [68]. Therefore, apoC-III not only mediates dyslipidaemia in insulin resistance, but also modulates intra-islet communication and β-cell fate. This offers new insight into the mechanisms that drive the progression from insulin resistance to overt β-cell failure in type 2 diabetes.

While these in vitro experimental observations offer intriguing, potential additional mechanisms linking apoC-III to ASCVD and diabetes risk, clinical evidence to support the postulated effects is as yet limited, and thus the translatability of the findings remains unclear.

### ApoC-III Inhibition, for Whom, When and How

ApoC-III inhibition has already been approved as a therapeutic strategy in the treatment of extreme hypertriglyceridemia where risk of recurrent acute pancreatitis is high [8]. However, this condition is rare, and it is the much wider application of this intervention to prevent cardiovascular disease that is a currently a major focus of attention. There is an increasing body of evidence, as summarised above, indicating that apoC-III is a prime candidate for intervention to lower ASCVD risk. The apoprotein is not only a major determinant of the plasma concentration of highly atherogenic TRL but also appears to have properties that promote atherogenesis beyond effects on lipoproteins. Further, metabolic investigations point to the benefits of suppressing apoC-III synthesis since common high-risk conditions such as obesity, insulin resistance and type 2 diabetes are associated with overproduction of the apoprotein. In this context, the availability of agents that directly inhibit *APOC3* expression and lower plasma apoC-III profoundly opens up an attractive therapeutic strategy.

### Clinical Experience with apoC-III Inhibitors

ApoC-III inhibitors have swiftly turned mechanistic and genetic insights into effective clinical therapies [69]. Both antisense oligonucleotides (e.g. olezarsen) and small interfering RNA (siRNA) agents (e.g. plozasiran) potently suppress hepatic apoC-III expression, reducing circulating apoC-III by up to 70%, as well as plasma triglycerides and remnant cholesterol by up to 50% [9–11]. The FDA’s approval of olezarsen in 2024 for familial chylomicronaemia syndrome (FCS) confirmed its clinical efficacy, with significant reductions in triglyceride levels and an 88% decrease in the incidence of pancreatitis, even in cases of complete LPL deficiency. Broader trials in multifactorial chylomicronaemia and mixed dyslipidaemia demonstrate consistent improvements in lipoprotein profiles, modest reductions in apoB-containing species and variable effects on LDL cholesterol.

Unlike previous agents such as pemafibrate and mixed omega-3 fatty acids, which failed to reduce cardiovascular events through triglyceride-lowering,, apoC-III inhibitors yield more favourable lipoprotein modulation [33, 70, 71]. Recent randomised controlled trials of olezarsen demonstrate reductions in triglycerides of up to 60%, reductions in apoC-III of over 70%, and significant improvements in VLDL-C, non-HDL-C, and apoB. At maximal doses, 91% of patients achieve triglyceride levels below 1.7 mmol/L [10, 12]. Adverse events are primarily limited to mild injection-site reactions, with no evidence of thrombocytopenia, hepatic or renal toxicity. A recent meta-analysis of ten trials confirmed consistent improvements in triglycerides, non-HDL-C and HDL-C, with a substantial reduction in the risk of acute pancreatitis and a safety profile similar to placebo [72].

Plozasiran, an investigational siRNA, has also demonstrated robust triglyceride-lowering effects in patients with severe hypertriglyceridaemia and mixed dyslipidaemia. Phase 2 trials have shown reductions of around 50% in triglyceride-rich lipoprotein particles, a shift towards larger LDL and HDL particles, and over 90% of patients have achieved triglyceride levels of less than 5.6 mmol/L [11]. These results were observed alongside mild-to-moderate adverse events, with no treatment discontinuations or deaths. Taken together, these results suggest that apoC-III inhibitors are a promising and mechanistically distinct option for high-risk patients with hypertriglyceridaemia.

### Impact of apoC-III Lowering on LDL Levels

Lowering apoC-III, either genetically or pharmacologically, effectively reduces TRLs and remnant cholesterol. However, its impact on LDL cholesterol varies depending on the context. When triglyceride levels are low to moderate, interventions that reduce TRLs tend to enhance LDL particle clearance [73]. However, as baseline triglyceride levels rise, LDL metabolism changes: there is a shift towards producing smaller, denser LDL particles, which are less easily cleared by the LDL receptor. This leads to elevated LDL particle concentration and enhanced atherogenicity. In individuals with severe hypertriglyceridaemia, LDL concentrations are paradoxically decreased due to accelerated LDL receptor-independent clearance driven by alternative catabolic pathways, likely involving the reticuloendothelial system [73]. When triglyceride levels are lowered from these very high initial levels, such as through apoC-III inhibition, fibrates, or other pharmacological means, the rate of receptor-independent LDL uptake decreases, as does the overall rate of LDL catabolism with the result that LDL particle concentration increases. Consequently, LDL cholesterol often rises in this situation. Therefore, the impact of reducing triglyceride levels on LDL levels depends not only on the initial triglyceride concentration, but also on the predominant LDL clearance pathway: receptor-dependent at low to moderate triglyceride levels and receptor-independent at high TG levels [73]. These complex changes emphasise that the effects of *APOC3* inhibition on LDL are not uniform, and will depend on the initial triglyceride level and the shift in LDL catabolic pathways. This explains the “*LDL paradox”* in severe hypertriglyceridaemia. Importantly, these mechanistic interactions must be carefully considered when designing clinical trials of TG-lowering therapies, as appropriate patient stratification by baseline triglycerides and recognition of the underlying LDL clearance mechanisms may profoundly influence both study outcomes and their clinical interpretation [73].

### Genetic Simulation Modelling: Quantifying Expected Benefit

Recently, Björnson et al. developed a TRL/remnant-specific polygenic score (PGS) in the UK Biobank cohort to model the expected impact of *APOC3* silencing on cardiovascular event rates [74]. By comparing individuals who were genetically predisposed to having either high or low TRL remnants (analogous to having an untreated or a pharmacologically treated condition), the results showed that having genetically lower TRL remnants produced striking reductions in plasma triglycerides (− 34%), remnant cholesterol (− 22.5%), non-HDL-C (− 7.5%) and apoB (− 6%), as well as a 28% lower lifetime risk of CHD events [74]. Notably, a 16–23 mg/dL difference in remnant cholesterol, which is within the range achieved by current *APOC3*-inhibitory therapies, is predicted to reduce the risk of major cardiovascular events by 25% over five years. These modelled benefits correspond to the changes observed with pharmacological siRNA and ASO agents, and this approach strengthens causal inference by mirroring the differences in lipoprotein profiles and events in both drug-treated subjects and those with a low genetic risk.

### Patient Selection: Who Stands to benefit?

Initiation of apoC-III inhibition is likely to be warranted in several high-risk situations, although confirmation from ongoing prospective clinical outcome studies is essential (Table 1). Nevertheless, current evidence suggests that this approach may be considered:

**Table 1:** Potential high-risk conditions for anti-CIII therapy

Patient type	Indication	Questions
Secondary prevention on statin	Residual risk of CVD event	Will risk reduction be proportionate to decrease in TRL/remnant concentration?
Type 2 diabetes at high risk of CVD	High CVD risk due to elevated TG/remnants ApoC-III overproduction	Does apoC-III lowering reduce CVD risk? Does CIII lowering improve glucose control?
Chronic kidney disease at high CVD risk	High CVD risk due to elevated TRL/remnants ApoC-III decreased clearance	Is apoC-III lowering effective in CKD related CVD risk?
High CVD SCORE2 with moderate HTG TG 1.7 to 5.0 mmol/l	High CVD risk due to elevated TG/remnants	What is value of apoC-III lowering in high-risk primary prevention? Does CIII lowering have benefits beyond TG reduction due to direct anti-atherosclerotic effects?
Severe HTG TG > 5.0 mmol/l or > 10.0 mmol/l (definitions vary)	Acute pancreatitis risk. CVD risk	What is the relationship of TG to CVD risk at very high TG levels? Does the increase in LDL on apoC-III lowering offset any CVD benefit of TG reduction?
Familial chylomicronemia syndrome (FCS due to LPL deficiency) Multifactorial chylomicronemia syndrome (MCS)	Severe TG/TRL elevation with high risk of recurrent acute pancreatitis can be alleviated by anti-apoCIII therapy via LPL independent mechanism.	Can those at high risk of first acute pancreatitis event be identified (especially MCS) and anti-CIII agents used prophylactically?

- In people at high ASCVD risk with moderate to severe hypertriglyceridemia when traditional lipid-lowering therapies such as statins, or omega-3 fatty acids fail to adequately control triglycerides and remnant cholesterol levels.​​.
- In patients with Type 2 diabetes where elevated apoC-III is a key component of the dyslipidemia and targeting the protein may offer a specific means of correcting the atherogenic lipoprotein pattern.
- In patients with recurrent pancreatitis or markedly elevated triglyceride levels, particularly those with underlying genetic defects affecting TRL metabolism such as FCS.​​.
- When residual atherogenic risk persists, particularly in those with established cardiovascular disease, despite optimal standard therapy, manifested by elevated non-HDL cholesterol or apoB in the setting of well-controlled LDL cholesterol.​​.

While outcome data from large clinical trials are not yet available, the consistent reductions in triglycerides, remnant cholesterol, and episodes of acute pancreatitis observed with multiple apoCIII–targeted therapies point to a promising therapeutic role in these settings. Final validation requires forthcoming studies to demonstrate cardiovascular event reduction, but the mechanistic and simulation evidence together make it increasingly likely that apoC-III inhibition will become a key adjunctive therapy for patients with significant residual metabolic risk.

### How: Therapeutic modalities, and Combinations

The main active ingredients, olezarsen (an antisense oligonucleotide) and plozasiran (a siRNA), are administered on a monthly to quarterly schedule. Safety and tolerability have been favourable, with Mendelian randomisation and clinical data showing no major adverse effects on liver function, glucose homeostasis or inflammation. Indeed, reductions in C-reactive protein (CRP) and white blood cell count post-therapy have been documented. Combination strategies integrating apoC-III inhibitors with statins or PCSK9 inhibitors are logical for patients with combined dyslipidaemia, as they allow the focus to be placed on both LDL- and remnant cholesterol levels.

## Conclusion

ApoC-III has emerged as a key regulator of TRL metabolism and an important, independent cardiovascular risk factor. Numerous clinical and metabolic studies have shown a strong positive correlation between plasma apoC-III levels and plasma triglyceride concentrations (Fig. 1). Kinetic studies using stable isotope tracers have transformed our understanding of this process by revealing the evidence that apoC-III levels causally influence TRL metabolism and thereby contribute substantially to cardiovascular disease risk.

The mechanisms by which apoC-III exerts its effects are multifaceted and include the inhibition of LPL, the impairment of hepatic remnant clearance, and via direct proatherogenic actions. These include promoting the retention and modification of lipoproteins in the artery wall, inducing inflammation, and affecting coagulation. The identification of distinct clearance rates and metabolic effects among apoC-III glycoforms adds an extra layer of complexity to this regulatory system. Although substantial progress has been made, important questions concerning tissue-specific functions and the relative importance of apoC-III glycoforms remain. Ongoing clinical trials of antisense oligonucleotides and siRNAs will provide crucial data on cardiovascular outcomes and safety. As clinical experience increases, the inhibition of apoC-III is poised to transform the management of triglycerides and remnant lipoproteins by offering personalised, timely cardiovascular risk reduction to high-risk patients.

## Key References

- Tardif JC, Karwatowska-Prokopczuk E, Amour ES, Ballantyne CM, Shapiro MD, Moriarty PM, et al. Apolipoprotein C-III reduction in subjects with moderate hypertriglyceridaemia and at high cardiovascular risk. Eur Heart J. 2022;43(14):1401-12. 10.1093/eurheartj/ehab820.
  - ○ This large clinical trial demonstrated that pharmacologic reduction of apoC-III reduced plasma triglycerides in individuals with moderate hypertriglyceridemia and high cardiovascular risk, providing evidence for the benefit of apoC-III inhibition in clinical practice.
- Kraaijenhof JM, Peletier MC, Nurmohamed NS, Hovingh GK, Alexander VJ, Tsimikas S, et al. Plasma reduction of apolipoprotein C-III with olezarsen leads to significant reductions in postprandial triglyceride levels: Results from a randomized trial. Eur J Prev Cardiol. 2025. 10.1093/eurjpc/zwaf478.
  - ○ This randomized trial showed that treatment with olezarsen, an antisense oligonucleotide targeting APOC3, substantially lowers postprandial triglyceride levels, underscoring the efficacy of specific apoC-III inhibition for triglyceride management.
- Ballantyne CM, Gaudet D, Rosenson RS, Hegele RA, Zhou R, Melquist S, et al. Effect of Targeting ApoC-III With Plozasiran on Lipoprotein Particle Size and Number in Hypertriglyceridemia. J Am Coll Cardiol. 2025;85(19):1839-54. 10.1016/j.jacc.2025.03.496.
  - ○ This study assessed the effect of the apoC-III inhibitor plozasiran on lipoprotein particle size and number in patients with hypertriglyceridemia, offering further clinical mechanistic data supporting the use of apoC-III targeting therapies.
- Bergmark BA, Marston NA, Prohaska TA, Alexander VJ, Zimerman A, Moura FA, et al. Olezarsen for Hypertriglyceridemia in Patients at High Cardiovascular Risk. N Engl J Med. 2024;390(19):1770-80. 10.1056/NEJMoa2402309.
  - ○ This pivotal trial evaluated olezarsen for hypertriglyceridemia in high-risk patients, demonstrating significant triglyceride and apoC-III lowering, helping validate the therapeutic approach for cardiovascular risk reduction.
- Taskinen MR, Bjornson E, Matikainen N, Soderlund S, Ramo J, Ainola MM, et al. Postprandial metabolism of apolipoproteins B48, B100, C-III, and E in humans with APOC3 loss-of-function mutations. JCI Insight. 2022;7(19). 10.1172/jci.insight.160607.
  - ○ This mechanistic study elucidated the effects of APOC3 loss-of-function mutations on postprandial metabolism of key apolipoproteins, providing strong genetic and metabolic support for targeting apoC-III in humans.
- Bjornson E, Packard C, Adiels M, Leeper NJ, Hellawell J, Gummesson A, et al. Genetic modelling of triglyceride-rich lipoprotein/remnant lowering mimics APOC3-silencing and predicts clinically relevant coronary heart disease event reductions. Eur J Prev Cardiol. 2025. 10.1093/eurjpc/zwaf657.
  - ○ This genetic modeling work predicted that triglyceride-rich lipoprotein (TRL)/remnant lowering by APOC3-silencing could yield clinically meaningful reductions in coronary heart disease risk, supporting the translational importance of APOC3 inhibition.

## Data Availability

No datasets were generated or analysed during the current study.
